# Lifestyle Subgroups and its Relationship with the Incidence of Hypertension in the Population of Azar Cohort: A Latent Class Analysis

**DOI:** 10.34172/hpp.44320

**Published:** 2026-06-06

**Authors:** Sahar Naghizadeh, Elnaz Faramarzi, Parvin Sarbakhsh, Hossein Akbari, Asghar Mohammadpoorasl

**Affiliations:** ^1^Department of Epidemiology, Faculty of Health, Iran University of Medical Sciences, Tehran, Iran; ^2^Liver and Gastrointestinal Diseases Research Center, Tabriz University of Medical Sciences, Tabriz, Iran; ^3^Department of Statistics and Epidemiology, Faculty of Health, Tabriz University of Medical Sciences, Tabriz, Iran

**Keywords:** Lifestyle, Hypertension, Physical activity, Sleep quality, Substance abuse

## Abstract

**Background:**

There is no study that simultaneously evaluates the relationship between lifestyle variables and the incidence of hypertension in the Iranian population. The aim of present study was to compare the incidence of hypertension across different lifestyle subgroups of the Azar cohort population identified via Latent Class Analysis.

**Methods:**

We used the data of 15,006 eligible participants across five follow-up periods. Seven observed variables were used to assess lifestyle behaviors as a latent variable. These indicators were smoking, substance abuse, alcohol consumption, secondhand smoke exposure, sleep quality, physical activity, and obesity. The analysis was performed in SAS 9.2 software.

**Results:**

Three-class and seven-class models were appropriate for females and males based on the indices for model selection and the interpretability of the model results, respectively. In females, 25.7%, 9% and 65.3% were at "low risk", "high risk" and "secondhand smoke exposure and poor sleep quality", respectively. In males, 13.3% and 3.6% were in the "smoker" and "high-risk" classes, respectively. In females and males (up to class 4), after adjusting for age and socioeconomic status, the prevalence and incidence of hypertension increased with the advancement of classes.

**Conclusion:**

Considering the characteristics of the identified classes and the occurrence of hypertension in each class, the main focus of lifestyle interventions can be placed on the most high-risk groups. Our findings suggest that poor physical activity, poor sleep quality, and obesity should be addressed as the main targets of lifestyle intervention strategies for preventing and controlling hypertension.

## Introduction

 Lifestyle is a complex and often generic concept and defined as patterns of behavior or patterns of behavioral choices influenced by socioeconomic conditions.^1^ Adopting the healthiest lifestyles would have a 55–71% lower risk of stroke, hypertension, CHD and heart failure.^2^ Lifestyle plays a pivotal role in increasing the incidence of hypertension.^3^ Lifestyle management recommendations have been established as the first-line strategy for the prevention and control of hypertension in adults.^4^ Lifestyle-related high-risk behaviors (eg, cigarette smoking, alcohol consumption, physical activity, overweight/obesity, sleep quality, substance use, exposure to secondhand smoke, diet) usually occurring simultaneously or in clusters.^5^ Accumulating high-risk lifestyle behaviors augments the risk of developing cardiovascular diseases; people with multiple risk behaviors have a poorer health status than those with only one risky behavior. Therefore, identifying individuals with multiple high-risk behaviors allows health policymakers to focus more on this group.^6^ Therefore, Lifestyle modification is vital to preventing hypertension; adequate control over this condition will save many lives.^7^ Evidence from high-risk behaviors indicates that gender attitudes and behaviors promote different patterns of healthy or unhealthy lifestyles among women and men. Gender has been recognized as an important factor that influences lifestyle habits and, consequently, the onset and course of chronic diseases.^8^ Males engage in more multiple health risk behaviors than females.^9^ Therefore, men and women should be analyzed separately in terms of the aggregation of high-risk behaviors and their association with the occurrence of diseases.

 Disease patterns in developing countries are switching from communicable to non-communicable diseases due to economic development and factors such as sedentary lifestyles^10^ and population aging.^11^ Non-communicable diseases like cardiovascular diseases, cancers, diabetes, and chronic respiratory diseases account for 74% of deaths worldwide.^12^

 Hypertension, defined as persistently elevated systolic blood pressure above 140 mmHg and/or diastolic blood pressure at least 90 mmHg and affects over 1.5 billion people worldwide.^4^ Hypertension represents a global public health challenge due to its high prevalence and relationship with cardiovascular diseases.^13^ It is the main modifiable risk factor for all-cause morbidity and mortality, targeted globally by the World Health Organization (WHO) for controlling non-communicable diseases.^14,15^ This condition is rising in prevalence, especially in developing countries.^13^ Also, it is responsible for 13.5% of deaths yearly.^16^

 As a developing country, Iran is undergoing an epidemiological transition due to factors such as population aging and changes in disease risk factors and patterns.^17,18^ Hypertension is the third leading cause of death in Iran.^19^ Despite scientific and technological advances and improved disease prevention measures, the overall prevalence of hypertension has increased in Iran, reported at 25% in 2017.^20^ The WHO holds that his condition accounted for about 5% of all deaths in Iran in 2020.^19^ Hypertension varies in prevalence from 19% to 40% across different regions of this country due to ethnic, cultural, socioeconomic, and lifestyle variations.^20,21^

 Latent class analysis (LCA) is a statistical means of identifying different subgroups of people in terms of a latent variable. LCA assigns people to subgroups based on the probability of fitting into a specific class in light of their response patterns in observed variables.^22^ Therefore, LCA takes a person-oriented approach,^23^ enabling us, for instance, to identify subgroups of people in terms of lifestyle.

 Behaviors related to lifestyle often coincide in specific patterns. Using LCA, we can simultaneously analyze the effects of variables related to lifestyle on the incidence of hypertension. Although lifestyle variables have previously been examined simultaneously, no such study has been conducted in the Iranian population to assess the association between lifestyle and the incidence of hypertension.^24-27^ Given the gap in the literature in this regard, we aimed to compare the incidence of hypertension across different lifestyle pattern subgroups of the Azar cohort population identified via LCA.

## Methods

###  Study design and setting

 The Azar Cohort Study, as a component of the larger PERSIAN (Prospective Epidemiological Research Studies in Iran) Cohort Study, examines the risk factors of common non-communicable diseases among Iranian adults.^28,29^ This study commenced in 2014 in the Shabestar region, East Azerbaijan province. This region is 2,630 km^2^, and the nearby Lake Urmia influences its climate. Its population is 124,499 people, with 48.5% living in urban areas. Most of the population (68%) is between 15 and 64. People aged 35 to 70 were invited to enter this study with the following criteria: permanent residence in the Shabestar region for at least nine months, written informed consent, and having at least one Azeri parent. Participants with mental or physical disabilities were excluded.^30-32^ The Azar Cohort Study has four phases: pilot, enrollment, follow-up, and re-evaluation. In the present study, we used the data of 15,006 eligible participants across five follow-up periods.

###  Variables

####  Smoking

 Smoking status was classified using three categories. Respondents were defined as (i) “non-smokers” if they had never smoked or had smoked less than 100 cigarettes in their lifetime, (ii) “ex-smokers” if they had smoked more than 100 cigarettes in their lifetime or had consumed one or more cigarettes per day but had not smoked for at least one year, or “smokers” if they currently smoked one or more cigarettes per day or used other types of tobacco (pipes, hookahs, etc.) at least once a week.

####  Substance use and alcohol consumption

 Substance use was designated as “no” or “yes”. If the participant had never used substances (e.g., heroin, amphetamines, barbiturates, cannabis, cocaine, hallucinogens, or opiates), they were classified in the “no” category. If they reported having used substances once or more, they were classified in the “yes” category. A similar scale was used to categorize participants regarding alcohol consumption.

####  Secondhand smoke exposure

 If the participants reported exposure to cigarette smoke for at least half an hour daily at work or home, they were classified in the “yes” group regarding exposure to secondhand smoke. Otherwise, they were classified in the “no” group.

####  Sleep quality

 In scoring the Total Sleep Quality Index (TSQI), a score of seven components is obtained, including the length of night sleep, sleep delay, habitual sleep efficiency, use of hypnotic medicine in the last week, sleep disorders (restless legs), disordered daytime functioning, and napping during the day. Component scores are summed to obtain a total score ranging from 0 to 17, with higher scores indicating inferior sleep quality. We classified the sleep quality across two categories: scores of 0-2 were “good,” while scores of 3 or more were classified as “poor or moderate.”

####  Physical activity

 Daily physical activity was measured with a 23-question self-reported questionnaire. Respondents reported their daily (24-hour) physical activities (light and heavy), rest, and sleep based on hours and minutes.^33,34^ The results were classified using the MET (Metabolic Equivalent of Task) scale in three categories based on the 25^th^ and 75^th^ percentiles: daily physical activity of ≥ 42.9 (METs/hour/Day) was classified as “good,” 37.8–42.8 (METs/hour/Day) was “moderate,” and ≤ 37.7 (METs/hour/Day) was “poor.” In the analysis of women, only two categories were used: “good and moderate” or “poor.”

####  Obesity status

 In the obesity status variable, people with a body mass index (BMI) of ≥ 30 kg/m^2^ were classified as “obese,” while people with a BMI of < 30 kg/m^2^ were classified as “normal or overweight.”

####  Socioeconomic status 

 Socioeconomic status (SES) was classified based on job categories, education levels, and family assets using principal component analysis (PCA). The categories included very high, high, middle, low, and very low based on SES quintiles.

####  Blood pressure

 During enrollment into the cohort study, subjects were considered to have hypertension if they reported being diagnosed with this condition. During the follow-up phases, all participants were telephoned annually and asked if they had been diagnosed with hypertension or used anti-hypertensive drugs. Those who answered positively were categorized as “yes”. Data from five annual follow-ups were used.

###  Statistical analysis

 For data analysis, we used LCA, a latent variable model that divides people into distinct subgroups in terms of latent variables using observed variables. All variables are categorical in this analysis; the main hypothesis is that membership in latent classes determines the pattern of individuals’ responses to observed variables. LCA, through different iterations for the number of identified classes of the latent variable and comparison of the frequency of the observed response patterns with the expected frequency, determines the best model and calculates the G2 statistic. Based on the G2 statistic, the AIC and BIC criteria can be calculated for model selection. Smaller values of these criteria indicate a more optimal balance of model fit. In our LCA, seven observed variables (i.e., indicators) were used to assess lifestyle behaviors as a latent variable. These indicators were smoking, substance use, alcohol consumption, secondhand smoke exposure, sleep quality, physical activity, and obesity. The analysis was performed using PROC LCA in SAS 9.2 software (SAS Institute Inc., Cary, NC, USA).

## Results

 More than half of the study participants were females (55.3%). In terms of age distribution, 16.7, 17.7, 17.9, 16.3, 13.9, 10.2 and 7.3 percent of participants were in the age categories of 35-39, 40-44, 45-49, 50-54, 55-59, 60-64 and 65-72 years, respectively.

 The frequency of lifestyle variables is given by gender in Table 1. Cigarette smoking, alcohol consumption, and substance use were more prevalent among males, whereas obesity and poor physical activity were more prevalent among females.

**Table 1:** Frequency of lifestyle variables by gender in the Azar cohort population

	**Males (n=6,712)**	**Females (n=8,294)**	**Total (n=15,006)**	***P*** **-value**
**Variable**	**n (%)**	**n (%)**	**n (%)**	
Cigarette smoking				
Non-smoker	3,205 (47.8%)	8,196 (98.8%)	11,401 (76.0%)	< 0.001
Ex-smoker	1,208 (18.0%)	38 (0.5%)	1,246 (8.3%)	
Smoker	2,299 (34.3%)	60 (0.7%)	2,359 (15.7%)	
Substance use				
No	6,459 (96.2%)	8,292 (100%)	14,751 (98.3%)	< 0.001
Yes	253 (3.8%)	2 (0%)	255 (1.7%)	
Alcohol consumption				
No	5,320 (79.3%)	8,267 (99.7%)	13,587 (90.5%)	< 0.001
Yes	1,392 (20.7%)	27 (0.3%)	1,419 (9.5%)	
Secondhand smoke exposure				
No	3,807 (56.7%)	4,128 (49.8%)	7,935 (52.9%)	< 0.001
Yes	2,905 (43.3%)	4,166 (50.2%)	7,071 (47.1%)	
Sleep quality status				
Good	2,768 (45.2%)	3,119 (40.6%)	5,887 (42.7%)	< 0.001
Moderate or poor	3356 (54.8%)	4,554 (59.4%)	7,910 (57.3%)	
Physical activity status				
Good	3,331 (49.6%)	1,676 (20.2%)	5,007 (33.4%)	< 0.001
Moderate	1,325 (19.7%)	3,678 (44.3%)	5,003 (33.3%)	
Poor	2,056 (30.6%)	2,940 (35.4%)	4,996 (33.3%)	
Obesity status				
Normal or overweight	4,969 (74.1%)	4,392 (53%)	9,361 (62.4%)	< 0.001
Obese	1,741 (25.9%)	3,895 (47%)	5,636 (37.6%)	

 Due to the very low prevalence of cigarette smoking, alcohol consumption, and substance use in women of the study population, as well as the difference in the prevalence of other variables across the two sexes, different LCA models were fitted for men and women.

###  Women’s model

 In the women’s model, only four dichotomous variables (secondhand smoke exposure, sleep quality status, physical activity status, and obesity status) were considered. Based on the four dichotomous variables, there were 16 possible response patterns. The comparisons of LCA models with different latent classes are presented in Table 2. We found that the three-class model was appropriate for females based on the indices for model selection and the interpretability of the model results.

**Table 2:** Comparison of latent class analysis (LCA) models with different numbers of latent classes based on model selection statistics in females.

**Number of latent classes**	**Log-likelihood**	**G2**	**AIC**	**BIC**	**df**	**Number of parameters estimated**	***P*** **-value**
1	-22054.1	68.97	76.97	105.07	11	4	< 0.001
2	-22033.5	27.71	45.71	108.92	6	9	< 0.001
3	-22019.7	0.12	28.12	126.45	1	14	0.729
4	-22019.7	0.11	38.11	171.55	-4	19	-
5	-22019.6	0.00	48.00	216.56	-9	24	-

Abbreviations: LCA = latent class analysis; AIC = Akaike information criterion; BIC = Bayesian information criterion.

 The results of the three-class LCA model for females are presented in Table 3. Accordingly, 25.7% and 9% of females were at low and high risk, respectively. Also, 65.3% of females were in the “secondhand smoke exposure and poor sleep quality” class.

**Table 3:** The three-class latent class analysis (LCA) model of lifestyle variables among females.

	**Latent class**		
	**Low-risk**	**Secondhand smoke exposure and poor sleep quality**	**High-risk**
**Latent class prevalence**	0.257	0.653	0.090
Item-response probabilities			
**Secondhand smoke exposure**			
No	0.644*	0.458	0.367
Yes	0.356	**0.542**	**0.633**
**Sleep quality status**			
Good	**0.592**	0.331	0.437
Moderate or poor	0.408	**0.669**	**0.563**
**Physical activity status**			
Good or moderate	**0.569**	**0.762**	0.024
Poor	0.431	0.238	**0.976**
**Obesity status**			
Normal or overweight	**0.658**	**0.520**	0.239
Obese	0.342	0.480	**0.761**

* Boldface fonts for Larger item-response probabilities to facilitate interpretation.

 Based on the established three-class model, the probability of membership in each class was calculated for each participant. Each participant was assigned to the class with the best probability. Table 4 presents crude and adjusted (for age and socioeconomic status) prevalence and incidence of hypertension in the three-class LCA model of lifestyle behaviors among females. Accordingly, after adjusting for age and socioeconomic status, the prevalence and incidence of hypertension increased with the advancement of classes.

**Table 4:** Prevalence and incidence of hypertension in the three identified latent classes of lifestyle behaviors among females

	**Prevalence (%)**		**Incidence (%)**	
**Latent class**	**Unadjusted **	Adjusted*	**Unadjusted**	Adjusted*
1	21.0%	20.8%	14.0%	15.6%
2	24.7%	24.7%	15.8%	17.5%
3	33.8%	34.1%	22.6%	26.2%

*: Adjusted for age and socioeconomic status.

###  Men’s model

 In the men’s model, five dichotomous variables (substance use, alcohol consumption, secondhand smoke exposure, sleep quality status, and obesity status) and two trichotomous variables (cigarette smoking and physical activity status) were considered. Accordingly, there were 288 possible response patterns. The comparison of LCA models with different numbers of latent classes is presented in Table 5. We found that the seven-class model was appropriate for males based on the indices for model selection and the interpretability of the model results.

**Table 5:** Comparison of latent class analysis (LCA) models with different numbers of latent classes based on model selection statistics in males.

**Number of latent classes**	**Log-likelihood**	**G2**	**AIC**	**BIC**	**df**	**Number of parameters estimated**
1	-30973.5	1929.8	1947.8	2009.1	278	19
2	-30287.4	557.6	595.6	725.0	268	29
3	-30194.7	372.1	430.1	627.6	258	39
4	-30169.1	320.9	398.9	664.5	248	49
5	-30149.9	282.6	380.6	714.4	238	59
6	-30138.6	259.9	377.9	779.8	228	69
7	-30120.8	224.3	362.3	832.3	218	79
8	-30114.8	212.4	370.4	908.5	208	89
9	-30105.8	194.4	372.4	978.7	198	99
10	-30104.2	191.1	389.1	1063.5	188	109
11	-30096.8	176.3	394.3	1136.8	178	119
12	-30090.9	164.5	402.5	1219.1	168	129

Abbreviations: LCA = latent class analysis; AIC = Akaike information criterion; BIC = Bayesian information criterion.

 The results of the seven-class LCA model for males are presented in Table 6. Accordingly, 13.3% and 3.6% of males were in the smoker and high-risk classes, respectively. Also, 29.3%, 16.3%, 23.1%, 5.7%, and 8.7% of males were in the second to sixth latent classes, respectively.

**Table 6:** The seven-class latent class analysis (LCA) model of lifestyle variables among males.

	**Latent Class**						
	**Smoker**	**Sleep problem**	**Smoker and sleep problems**	**Sleep problems and poor physical activity**	**Ex-smoker, secondhand smoke exposure, and sleep problems**	**Smoker, alcohol consumer, and sleep problems**	**High-risk(smoker, alcohol consumer, secondhand smoke exposure, poor physical activity, and obese)**
**Latent class prevalence**	0.133	0.293	0.163	0.231	0.057	0.087	0.036
**Cigarette smoking**							
Non-smoker	0.025	0.992*	0.000	**0.726**	0.229	0.000	0.079
Ex-smoker	0.213	0.000	0.237	0.274	**0.629**	0.074	0.197
Smoker	**0.762**	0.008	**0.763**	0.000	0.142	**0.926**	**0.724**
**Substance use**							
No	**0.998**	**0.999**	**1.000**	**0.997**	**1.000**	**0.709**	**0.691**
Yes	0.002	0.001	0.000	0.003	0.000	0.291	0.309
**Alcohol consumption**							
No	**0.789**	**0.963**	**0.823**	**0.918**	**0.593**	0.245	0.120
Yes	0.211	0.037	0.177	0.082	0.407	**0.755**	**0.880**
**Secondhand smoke exposure**							
No	**0.707**	**0.584**	**0.697**	**0.508**	0.000	**0.704**	0.286
Yes	0.293	0.416	0.303	0.492	**1.000**	0.296	**0.714**
**Sleep quality status**							
Good	**0.996**	0.452	0.017	0.485	0.360	0.365	**0.575**
Moderate or poor	0.004	**0.548**	**0.983**	**0.515**	**0.640**	**0.635**	0.425
**Physical activity status**							
Good	**0.498**	**0.625**	**0.539**	0.326	**0.602**	**0.494**	0.184
Moderate	0.160	0.152	0.218	0.281	0.129	0.168	0.254
Poor	0.342	0.223	0.243	0.393	0.269	0.338	**0.562**
**Obesity status**							
Normal or overweight	**0.778**	**0.852**	**0.756**	**0.584**	**0.687**	**0.873**	0.394
Obese	0.222	0.148	0.244	0.416	0.313	0.127	**0.606**

* Boldface fonts for larger item-response probabilities to facilitate interpretation.

 Based on the established model, the probability of membership in each class was calculated for each participant. Each participant was assigned to the class with the best probability. Table 7 presents the crude and adjusted (for age and socioeconomic status) prevalence and incidence of hypertension in the seven-class LCA model of lifestyle behaviors among males. After adjusting for age and socioeconomic status, the prevalence and incidence of hypertension increased with the advancement of classes up to class 4. However, the prevalence and incidence of hypertension among participants in the sixth class were the same as in the first class.

**Table 7:** Prevalence and incidence of hypertension in the seven identified latent classes of lifestyle behaviors among males

	**Prevalence (%)**		**Incidence (%)**	
**Latent class**	**Unadjusted **	**Adjusted**	**Unadjusted **	**Adjusted**
1	11.2%	11.2%	8.6%	9.0%
2	12.8%	12.8%	11.1%	11.8%
3	14.1%	13.9%	11.2%	11.8%
4	23.3%	23.4%	16.5%	17.8%
5	18.5%	18.5%	13.3%	13.9%
6	10.8%	10.9%	9.2%	9.3%
7	20.4%	20.4%	14.5%	15.5%

## Discussion

 This study aimed to determine subgroups of the Azar cohort population based on lifestyle patterns using the LCA method and compare the incidence of hypertension in lifestyle behavior subgroups. Considering the behavioral differences between men and women in Iranian society, the two sexes were investigated separately. Classes became different for women and men. Women were classified into three classes, whereas men were classified into seven classes. The model for women, based on lifestyle patterns, was defined as follows: a healthy (low-risk) lifestyle (25.7%), a moderate-risk lifestyle (secondhand smoke exposure and poor sleep quality) (65.3%), and a high-risk lifestyle (9.0%). The high-risk lifestyle included obese individuals with secondhand smoke exposure and poor sleep quality/physical activity. Men were classified into seven distinct classes based on lifestyle patterns: class 1 (13.3%) included smokers; class 2 (29.3%) included men with poor sleep quality; class 3 (16.3%) featured smokers with poor sleep quality; class 4 (23.1%) included people with poor sleep quality and physical activity; class 5 (5.7%) included ex-smokers exposed to secondhand smoke with poor sleep quality; class 6 (8.7%) included smokers and alcohol consumers with poor sleep quality; and class 7 (3.6%) included obese smokers with alcohol consumption, secondhand smoke exposure, and poor physical activity. One such study in Iran, conducted on 750 hypertensive patients over 50 years, identified Three classes of lifestyle patterns. About 14.4% of hypertensive patients were categorized in a low-risk class, 54.6% in an intermediate-risk class, and 31% in a high-risk class of lifestyle.^35^

 In the present study, the frequency of smoking, alcohol consumption, and substance use was higher among men than women, in line with Moradinazar et al.^30^ Similarly, Shen et al. recorded a higher prevalence of smoking and alcohol consumption in men than in women.^36^ On the other hand, in agreement with the findings of Ghanbari et al., we found obesity and poor physical activity to be more common among women than men.^35^

 In the present study, the highest prevalence and incidence of hypertension in the female model was related to the high-risk class (class 3). Zhang et al. demonstrated an association between secondhand smoke exposure and hypertension risk.^37^ Also, a study linked secondhand smoke exposure with hypertension among non-smokers (OR = 1.16).^38^ On the other hand, previous studies have shown that both short and long sleep durations are related to an increased risk of hypertension in most age groups.^39,40^ Ewunieet al. concluded that sedentary adults are 2.55 times more likely to suffer from hypertension than physically active adults.^41^ According to Wenzhen Li et al., the combined effect of low physical activity and high BMI is associated with the highest risk of hypertension.^42^ Obesity is one of the most critical determinants of hypertension. The chance of having hypertension in obese adults is two^43^ to three ^42^ times higher compared to those with normal BMI. Misuzu Fujita et al. demonstrated a stronger effect of obesity on the incidence of hypertension in women than in men.^44^ In the present study, women in the high-risk class accumulated all these risk factors, increasing the prevalence and incidence of hypertension.

 In the male model, the highest prevalence (23.4%) and incidence (17.8%) of hypertension were related to class 4 (poor physical activity and sleep quality). This class included almost a quarter of all men (23.1%), reflecting the necessity of paying more attention to this group. In this class, the incidence and prevalence of hypertension were significantly greater compared with class 3 (smokers with poor sleep quality). As poor sleep quality was present in both classes, these differences are most likely related to the effect of physical activity. On the other hand, the comparison of class 1 (smokers) with class 3 (smokers with poor sleep quality) showed that the addition of sleep problems led to an increase in the incidence and prevalence of hypertension. These two influential factors, i.e., poor physical activity and poor sleep quality, were aggregated in class 4. According to Belinda Hernández et al., poor sleep quality and physical activity are associated with a higher risk of chronic diseases, including hypertension.^45^ Yongbin Li et al. showed a significant correlation between physical activity and sleep.^46^ Merellano-Navarro et al. found that being a man and having appropriate physical activity improves sleep quality regardless of age.^47^ Also, one review concluded that moderate physical activity affects sleep quality more than intense physical activity in all age groups.^48^ Therefore, through interventions that increase physical activity, sleep quality can also be improved, theoretically reducing the incidence of hypertension in high-risk groups.

 After class 4, the highest incidence and prevalence of hypertension was related to class 7 (obese smokers with alcohol consumption, secondhand smoke exposure, and poor physical activity). A problematic behavior common to both these classes is poor physical activity. In 2018, the Physical Activity Guidelines Advisory Committee provided strong evidence on the protective effect of physical activity against hypertension based on a meta-analysis of 15 clinical trials.^49^ Also, the inverse relationship between physical activity and hypertension has been reported in different countries (Britain, China, Denmark, France, Italy, Korea, Saudi Arabia, and Thailand), consistent with our results.^50,51^ A study compared physical activity with other lifestyle interventions (weight loss, diet modification, smoking cessation, and moderation of alcohol consumption) and showed that among the recommended lifestyle changes, increased physical activity had broad benefits.^52^ Another important reason for the high incidence and prevalence of hypertension in class 7 is obesity. In this regard, a study concluded that overweight and obesity are significant and independent risk factors for hypertension; this relationship was significant after adjusting for confounding factors such as age, gender, smoking, healthy diet, and physical activity.^53^ For every 3 kg/m^2^ increase in BMI, the risk of hypertension increases by 50% in men and 57% in women.^54^ Regular physical activity and weight control can reduce the risk of hypertension, and physical activity’s protective effect remains the same in both sexes regardless of obesity levels.^55^

 Among men, by comparing class 2 (poor sleep quality) and class 3 (smokers with poor sleep quality), adding smoking to class 3 did not result in a higher incidence of hypertension. Regarding the effect of smoking on blood pressure, prior studies have provided conflicting results. Smoking causes an acute and transient increase (about 15 minutes) in blood pressure.^56^ However, the chronic effect of smoking on hypertension is controversial.^57^ Another study showed that, only in older men, blood pressure was significantly higher in smokers than in non-smokers.^58^ Also, class 3 (smokers with poor sleep quality) featured a lower incidence and prevalence of hypertension than class 5 (ex-smokers with secondhand smoke exposure and poor sleep quality). In this regard, Guoju Li et al. revealed lower blood pressure levels in smokers than in non-smokers and ex-smokers.^57^ A meta-analysis showed that smoking is associated with lower blood pressure and a decreased prevalence of hypertension.^59^ One explanation may be the lower BMIs in smokers and higher BMIs in ex-smokers^60^ since a significant interaction exists between BMI, smoking, and blood pressure among men.^58^ However, these findings should not distract attention from the known harms of smoking.^61^ The inverse relationship between smoking and hypertension and the higher prevalence of hypertension in ex-smokers may be because doctors are more likely to advise people with hypertension to quit smoking. In fact, ex-smokers are more aware of their blood pressure than non-smokers, pushing them toward quitting.^62^ On the other hand, a cohort study showed that smoking cessation significantly reduces blood pressure.^63^ Smoking cessation is strongly recommended for its well established health benefits. Importantly, strategies and approaches to avoid weight gain following smoking cessation should be implemented.^4^

 The incidence and prevalence of hypertension were lower in class 6 than in class 3. As the difference between these two classes was in the presence of alcohol consumption in class 6, our findings reflect a protective role of alcohol against the occurrence of hypertension. Similarly, Won Kim Cook et al. found a lower hypertension prevalence in smokers and alcohol consumers.^64^ A systematic review of 32 randomized controlled trials concluded that low and moderate doses of alcohol reduce blood pressure, while high doses have a biphasic effect on blood pressure, reducing it in the first 12 hours but increasing it after 13 hours.^65^ Naghipour et al. found no independent relationship between alcohol consumption or smoking and hypertension risk.^21^ on the other hand a synergistic effects were observed in a study by adding alcohol consumption on smoking models in men and women.^66^ Drinking and smoking behaviors generally occur together. Alcohol consumption can affect the relationship between smoking and blood pressure, whereas the relationship between alcohol consumption and blood pressure did vary by smoking status.Thus, the synergistic effects remained unclear.^67^ Among white males, one study concluded that large amounts of alcohol are an independent risk factor for hypertension, but low to moderate alcohol consumption is not associated with a higher incidence of hypertension.^68^ Min-GyuYoo et al. linked moderate and heavy alcohol consumption with the risk of hypertension in men.^69^ In our study, the classification of alcohol consumption was such that people who had ever experienced alcohol consumption were considered as part of the “yes” answer, which includes a large share of alcoholics. Therefore, the lower incidence and prevalence of hypertension in class 6 may be related to the roles of alcohol and smoking. In this regard one study has shown that the combine reduction in alcohol consumption and tobacco smoking was associated with reduction in hypertension.^70^ Overall, the findings regarding the effects of smoking and alcohol on hypertension are diverse and sometimes contradictory.

## Strengths and limitations

 We ran LCA models for women and men separately to maximize statistical power since risky lifestyle behaviors follow different patterns between the sexes. The large sample size and the use of information from five follow-up periods were also among the study’s strengths. However, this study also had limitations. As the age at entry into the study was 35-70 years, comparisons with studies conducted on adults of all ages might be limited. Furthermore, the level of smoking, alcohol consumption, and secondhand smoke exposure was not considered.

## Conclusion

 The present study determined subgroups of the Azar cohort population based on lifestyle patterns using LCA and compared the incidence and prevalence of hypertension between these subgroups. This study provides important information on lifestyle intervention strategies to minimize the burden of hypertension. Identifying concurrent high-risk behaviors in an at-risk population can lead to simultaneous interventions as an effective means of preventing disease by addressing clusters of high-risk behaviors. Considering the characteristics of the identified classes and the prevalence and occurrence of hypertension in each class, the main focus of lifestyle interventions can be placed on the most high-risk groups. Our findings suggest that poor physical activity, poor sleep quality, and obesity should be addressed as the main targets of lifestyle intervention strategies for preventing and controlling hypertension. Increased physical activity is vital in controlling BMI and improving sleep quality, thereby preventing hypertension.

## Competing Interests

 The authors declare no competing interests.

## Ethical Approval

 This cross-sectional study was approved by Ethics Committee in Tabriz University of Medical Sciences (code: IR.TBZMED. REC.1401.099). All participants filled out and signed informed consent forms, and they had the right to leave the study whenever they want at any time.
